# Prediction of Recurrence in Cervical Cancer Using a Nine-lncRNA Signature

**DOI:** 10.3389/fgene.2019.00284

**Published:** 2019-04-03

**Authors:** Yu Mao, Lixin Dong, Yue Zheng, Jing Dong, Xin Li

**Affiliations:** Department of Oncology, First Hospital of Qinhuangdao, Qinhuangdao, China

**Keywords:** cervical cancer, lncRNA, prognosis, GEO, TCGA

## Abstract

**Background and Objective:**

As a common cancer type in women, cervical cancer remains one of the leading causes of cancer-associated mortalities word wide. Recent evidence has demonstrated the regulatory role of a large number of long non-coding RNAs (lncRNAs) in cervical cancer. Here, we aimed to identify new biomarkers that related with the recurrence through comprehensive bioinformatics analysis.

**Methods:**

Firstly, we collected online lncRNA expression data of cervical cancer patients which were divided into training, validation, and test set. Then we developed a nine-lncRNA signature from training set by conducting LASSO Cox regression model along with 10-fold cross validation. The prognostic value of this risk score was validated in all the three sets using Kaplan–Meier analysis, C-index, time-dependent ROC curves and dynamic AUC. Biological function of these lncRNAs in cervical cancer cells were evaluated by performing gene ontology biological process enrichment and Kyoto Encyclopedia of Genes and Genomes signaling pathways analysis.

**Results:**

According to the results, a higher predict accuracy was observed in the nine-lncRNA signature than that of FIGO stage in all the three sets. Stratified analysis also demonstrated that the nine-lncRNA signature can predict the recurrence of cervical cancer within FIGO stage. The potential mechanisms underlying the nine-lncRNAs from the signature were also identified according to the gene enrichment analysis.

**Conclusion:**

In the present article, we provided a reliable prognostic tool to facilitate the individual management of patients with cervical cancer after treatment.

## Introduction

As the second most common cause of female cancer-associated mortalities worldwide, cervical cancer ranks the fourth most frequently diagnosed cancer (Jemal et al., 2011; Torre et al., 2015). Previous studies demonstrated that cervical cancer was closely associated with human papillomavirus (HPV) infection (Walboomers et al., 1999; Castellsagué et al., 2006). Evidence suggested that HPV mediated the genomic instability and somatic mutations which play important roles in the pathogenesis of cervical cancer (Cancer Genome Atlas Research Network et al., 2017). Despite great advancement achieved in the standard treatment such as surgery, radiotherapy, and chemotherapy, the prognosis for patients with cervical cancer remains poor (Fuller et al., 2007). Hence, identification of the new suitable prognosis biomarkers are critical for cervical cancer. Recent studies have showed that genomic factors could be the indicators for the prognosis of cervical cancer (Mao et al., 2018a).

Previous studies based on the gene expression data have identified a series of gene signature which was used as recurrence predictive model. Huang et al. (2012) has identified a 7-gene signature which associated with the relapse and survival in patients with early stage cervical carcinoma. Lee et al. (2013) selected 12-genes and constructed a prognostic score model for recurrence prediction. However, limited number of studies investigated whether the long non-coding RNA (lncRNA) signature can predict the recurrence and disease free survival time of cervical cancer.

As the RNA transcripts longer than 200 nucleotides, lncRNAs lack the ability of generating protein (Gupta et al., 2010). However, they can still contribute to the modulation of tumor progression associated biological processes via chromatin remodeling, transcription and post-transcriptional processing (Tano and Akimitsu, 2012; Wang et al., 2014). In addition, recent studies have showed the association between lncRNAs and survivals of human cancers, such as prostate cancer, breast cancer and gastric cancer (Hu et al., 2014; Zhu et al., 2016; Li et al., 2018).

The Gene Expression Omnibus is an international public repository that archives and freely distributes microarray, next-generation sequencing, and other forms of high-throughput functional genomic data sets (Kodahl et al., 2014; Clough and Barrett, 2016). The Cancer Genome Atlas (TCGA) is a large-scale cancer genome project which provides researchers with multi-dimensional maps of the key genomic changes and clinic-pathological information in 33 types of cancer (Collins and TCGA Project Team, 2007). Hence, we downloaded the LncRNA data from the GEO and performed lncRNA profiling on cervical cancer patients. Finally, a prognostic, nine-lncRNA signature for cervical cancer was constructed from the training set of GEO and its predictive accuracy was further validated two independent validation sets.

## Materials and Methods

### Data Source

As we have previously depicted, we firstly downloaded the “MINiML formatted family file(s)” of GSE44001 datasets from GEO which has the microarray data of 300 patients with cervical cancer and then processed these data sets using R to generate the lncRNA expression matrix. The clinical information (recurrence status, disease free survival time, and FIGO stage) of 300 patients was also extracted and patients were randomly assigned to a training set (*n* = 150) and an internal validation set (*n* = 150). RNA sequencing (RNA-seq) and corresponding clinical data (recurrence status and disease free survival time) were downloaded from the publicly available TCGA database. After excluded those without complete clinical and survival information, a number of 49 patients with cervical cancer were enrolled into the external test set. Each lncRNA expression level was determined by the value of Reads Per Kilobase of exon model per Million mapped reads (RPKM) (Mao et al., 2018b, 2019b).

### Microarray Data Analysis and lncRNA Signature Construction

Data preprocessing was performed according to our previous published study (Mao et al., 2018b,c, 2019a). After quantile normalization and log2-scale transformation, we delineated the box plot (Supplementary Figure S1). Then we performed cox regression analysis with Least Absolute Shrinkage and Selection Operator (LASSO), which is a parameter selection method that manage high-dimensional regression variables with no prior feature selection step by shrinking all regression coefficients and forcing many variables to be exactly zero (Tibshirani, 1997; Mao et al., 2019b). To achieve variable selection and shrinkage, we put the normalized lncRNA expression data into LASSO Cox regression. During this process, the penalty regularization parameter λ was chosen via the cross-validation routine with an *n*-fold = 10 by using R package “glmnet” (Friedman et al., 2010). The value of lambda.min, the lambda value giving minimum mean cross-validated error was calculated using R. By applying the lambda.min, a nine-lncRNA signature was identified based on the expression of lncRNAs weighted by the coefficients from LASSO penalized regression. Then the score of each sample was calculated according to expression levels of the RNAs (Expi) and LASSO coefficients (L_i_).

$$\mathrm{Risk score}=\sum_{i=1}^{n}{\mathrm{Exp}}_{i}\times L_{i}$$

The resulting score allowed the division of patients into two classes, namely the high-risk group and low risk group based on the median risk score value.

### Prognostic Signature Validation

Firstly, Kaplan–Meier and Chi-square analysis was performed using Graphpad prism. C-index was calculated using R with Package “survival” (Therneau and Grambsch, 2000). After that, a time-dependent ROC (receiver operating characteristic) curves along with the dynamic area under the time specific ROC curves (dynamic -AUC) was obtained by using R with Package “risksetROC” (Heagerty et al., 2000; Heagerty and Zheng, 2005).

### Functional Annotation of lncRNA Target Genes

Identifying target genes of lncRNAs is an important step in studying the function of lncRNA in cervical cancer. In this study, we first predict the target genes of lncRNAs in the signature by using the starBase v2.0 (Yang et al., 2011; Li et al., 2014). Then these genes were put into gene ontology (GO) biological process enrichment, Kyoto Encyclopedia of Genes and Genomes (KEGG), and REACTOM^1^ signaling pathways analysis. The enrichment analysis results, including enrichment score, the count of genes enriched in the terms and false discovery rate were shown as pictures which plotted using R with Package “ggplot2” (Wickham, 2015). The potential relationship among these target genes was analyzed using Search Tool for the Retrieval of Interacting Genes (STRING) which is an online tool designed to evaluate the protein–protein interaction (PPI) information (Damian et al., 2015). The PPI network were then plotted.

Moreover, we identified the related genes of the nine-lncRNAs in the signature by calculating pearson correlation coefficient between lncRNAs and mRNAs using TCGA datasets as previously depicted (Mao et al., 2018b,c, 2019a). Genes with pearson correlation coefficient >0.60 or <−0.40 was considered as associated with genes in the signature and was enrolled into the analysis. After that, these genes were also put into functional enrichment and pathway analysis which was then visualized using Cytoscape software with ClueGO and CluePedia (Shannon et al., 2003; Bindea et al., 2009).

## Results

### Prognosis Related lncRNA Identification and Signature Generation

**FIGURE 1:**
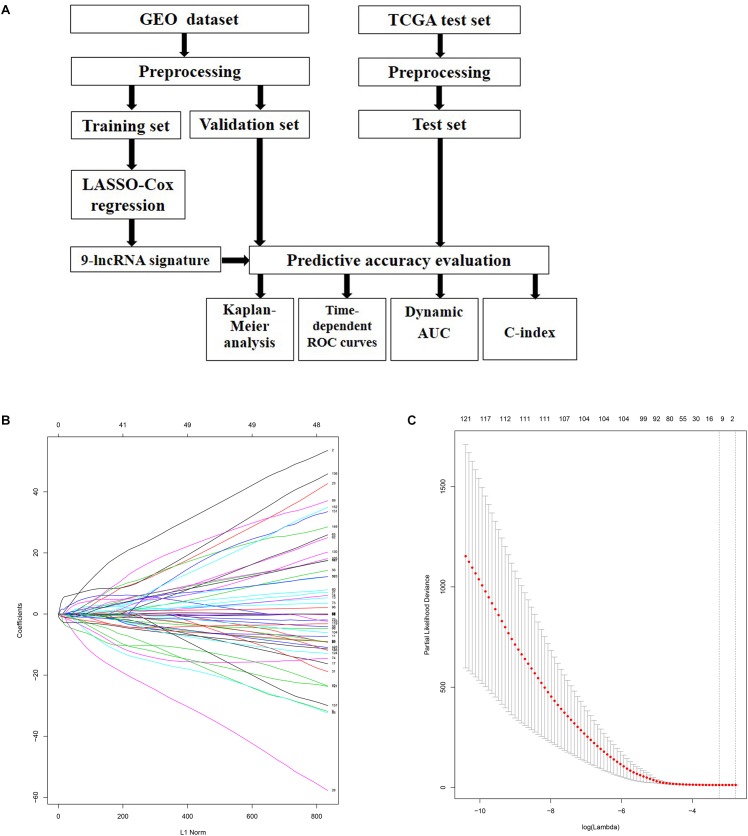
Identification of prognosis related lncRNAs using LASSO regression model. **(A)** Flowchart of the whole analysis process. **(B)** LASSO coefficient profiles of the lncRNAs associated with the disease free survival of cervical cancer. **(C)** Plots of the cross-validation error rates. Each dot represents a lambda value along with error bars to give a confidence interval for the cross-validated error rate. The top of the plot gives the size of each model. The vertical dotted line indicates the value with the minimum error and the largest lambda value where the deviance is within one SE of the minimum.

A flowchart which depicted the whole process of our analysis was firstly plotted in Figure 1A. Samples in GEO datasets were firstly quantile normalized and the distributions for the dataset of lncRNA profiles in each patient was shown as box plot using the R software package (Supplementary Figure S1). 300 patients from the GSE44001 cohort were randomly divided into a training cohort (*n* = 150) and internal validation cohort (*n* = 150). LASSO Cox regression model along with 10-fold cross validation was performed to analyze the expression data in the training cohort (Figure 1B). According to the results, a set of nine-lncRNAs along with their coefficients were identified and included into a risk score formula. Hence, we developed a nine-lncRNA signature based on their expression level and coefficients. Risk score = (3.31562585 ∗ ATXN8OS) + (0.13987057 ∗ C5orf60) + (−0.43216636 ∗ DIO3OS) + (−0.92247218 ∗ EMX2OS) + (1.13309789 ∗ INE1) + (−4.48055889 ∗ KCNQ1DN) + (−0.08067727 ∗ KCNQ1OT1) + (−0.09737496 ∗ LOH12CR2) + (−0.66622831 ∗ RFPL1S) (Figure 1C).

**FIGURE 2:**
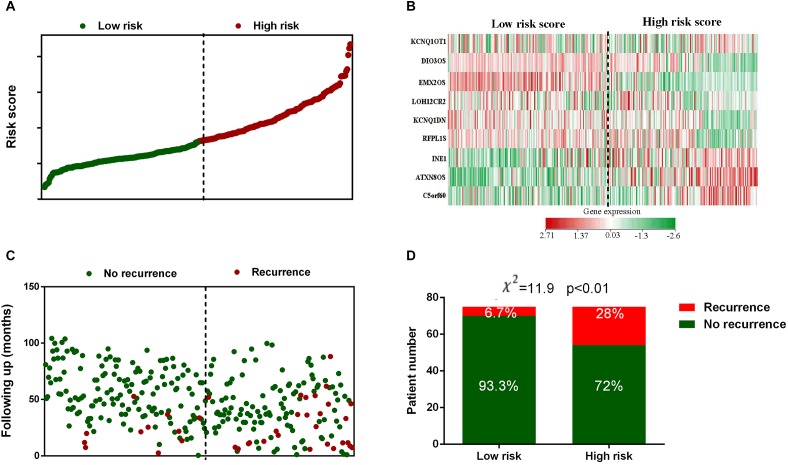
Risk score distribution, gene expression profile and recurrence data of the GEO training set. **(A)** The distribution of each patients’ risk score from the GEO training set. **(B)** Heat map of the lncRNAs in prognostic signature. **(C)** The outcome of recurrence status and time of patients in GEO training set. **(D)** Recurrence rate in low- and high-risk score groups. The black dotted line represents the optimum cutoff dividing patients into low-risk and high-risk groups.

The risk score for each patient in the training set was calculated and plotted in Figure 2A. Besides, the corresponding heatmap of lncRNA expression level in the signature was also presented. Figure 2C showed the distribution of disease free survival time and recurrence status of each patient which ranked according to the score in Figure 2A. According to Figure 2B,C, three lncRNAs in the signature had positive coefficients including ATXN8OS, C5orf60, and INE1 which revealed that higher expression level of these lncRNAs was associated with shorter disease free survival while the other six lncRNAs including DIO3OS, EMX2OS, KCNQ1DN, KCNQ1OT1, LOH12CR2, and RFPL1S had negative coefficients which indicated that their expression level was negatively related with the possibility of cervical cancer recurrence. Moreover, Chi-square analysis showed that the recurrence rate in high risk group was significant higher than low risk group (Figure 2D).

### Validation of the lncRNA Signature’s Survival Predict Accuracy

**FIGURE 3:**
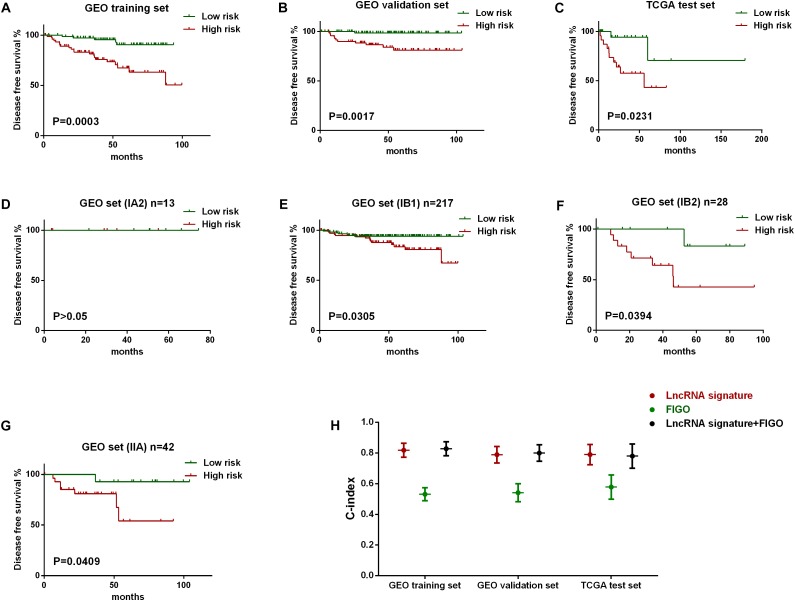
Evaluation of the predict efficiency of nine-lncRNA signature using Kaplan-Meier analysis along with log rank p and C-index. Kaplan-Meier curves were used to visualize and compare the disease free survival of low-risk group versus high-risk group in GEO training set **(A)**, GEO internal validation set **(B)**, TCGA test set **(C)**. Stratified analysis was further performed to evaluate the predictive efficiency of the lncRNA signature within different FIGO stages, including IA2 stage **(D)**, IB1 stage **(E)**, IB2 **(F)**, and IIA stage **(G)**. The C-index value of three variables in all three data sets was also plotted **(H)**.

The robustness of the lncRNA signature was tested by evaluating their ability to classify the high-risk group and low risk group in all the three datasets. The gene signature based risk score for each patient was firstly calculated. Then patients were divided into high- or low-risk group according to the median value. Kaplan–Meier curves were plotted, along with log rank *p*-test, to compare the disease free survival of the two groups. According to the results, significant differences in Kaplan–Meier survival analysis were observed in high- and low- risk group separated by the lncRNA signature in GEO training (Figure 3A) and validation set (Figure 3B). Similar outcomes were also found in external validation set (TCGA test set) (Figure 3C).

Besides, stratified analysis was further performed to evaluate the predictive efficiency of the lncRNA signature within FIGO stages. Figure 3E–G showed that the lncRNA signature can predict the tumor recurrence in patients of different FIGO stage, except for those in IA2 stage as a results of the limited number (Figure 3D). We also evaluate the predict accuracy of the lncRNA signature by using C-index which showed that the C-index value of lncRNA signature was higher than FIGO and a new variable combing both has the highest value than either alone (Figure 3H).

### Further Evaluation of the Nine-lncRNA Signature Predictive Efficiency Using Time-Dependent ROC Curves and Dynamic AUC

**FIGURE 4:**
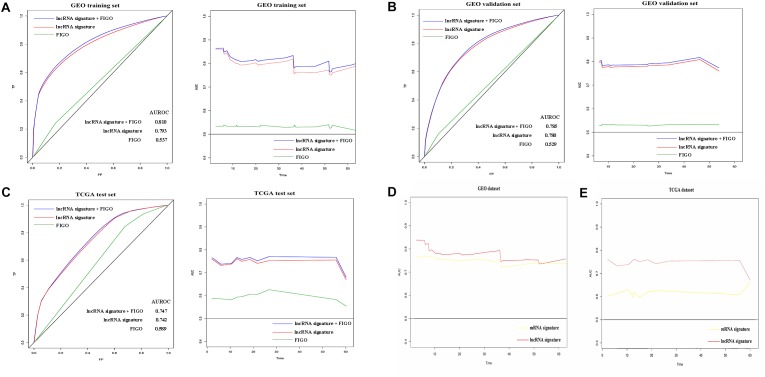
Prognostic value evaluation of three variables using time specific ROC curves and dynamic AUC lines. The time-dependent ROC curves on the 12th month of follow up and the dynamic AUC lines were plotted for patients in GEO training set **(A)**, GEO validation set **(B),** and TCGA test set **(C)**. The dynamic AUC lines of two signatures in GEO **(D)** and TCGA **(E)** dataset.

Next, we tried to determine the sensitivity and specificity of the predictive model and the changes of predictive accuracy over time. Hence, we introduced time-dependent ROC (receiver operating characteristic) curves and dynamic AUC to assess the predictive accuracy of lncRNA signature, FIGO stage and a new variable combined both. Firstly, we plotted the time-dependent ROC curves and calculated the corresponding AUC on the 12th month of follow up. As shown in Figure 4, predict accuracy of the new variable which combined both the signature and FIGO was better than either alone on the 12th month of follow up in all three sets. In addition, the lncRNA signature has a better predictive accuracy than FIGO stage in all the three subsets (Figure 4).

Subsequently, the dynamic area under curves (dynamic -AUC) at continuous time was calculated and depicted as the line chart. Results showed that the lncRNA signature combined with FIGO stage has a higher dynamic AUC level than either alone. The lncRNA alone also showed high accuracy with the dynamic AUC estimates exceeding 0.75 which was much better than FIGO stage alone. Similarly, the nine-lncRNA signature showed better predict accuracy than FIGO stage in the GEO validation set and TCGA test set as shown in Figure 4B,C. Hence, the lncRNA signature could predict the recurrence of cervical cancer patients with high efficiency.

Furthermore, we compared the recurrence predict performance of the nine-lncRNA signature with a 12-mRNA signature which was established in a previously published article (Lee et al., 2013). Results showed that similar predict accuracy was observed between the two signatures in GEO datasets (Figure 4D). However, the nine-lncRNA signature showed a better predict ability than the 12-gene signature in TCGA dataset (Figure 4E).

### Functional Annotation of lnRNA Associated Genes

**FIGURE 5:**
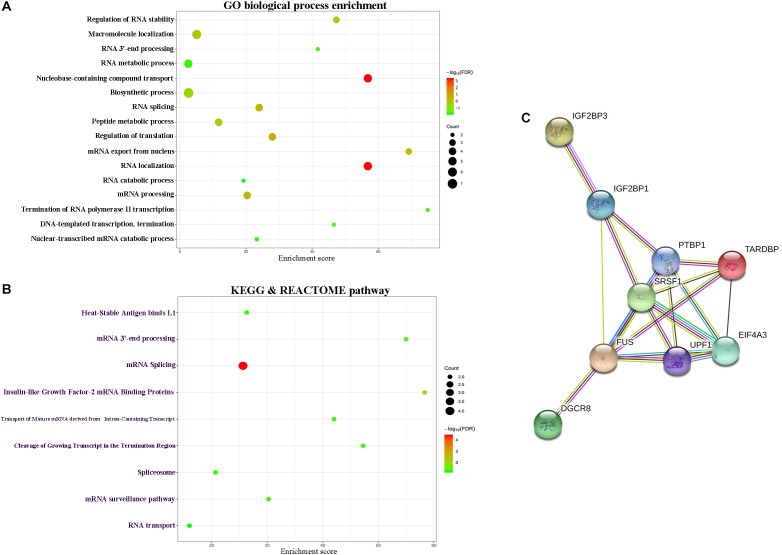
Functional enrichment analysis depicted the biological pathways and processes associated with lncRNAs in the signature. The results of GO biological process enrichment **(A)**. KEGG and REACTOM signaling pathways analysis **(B)**. PPI network of the target genes **(C)**.

In order to further describe the function of lncRNAs in the signature, we performed the function annotation and enrichment analysis. Studies on lncRNA have demonstrated that lncRNAs usually act as competing endogenous RNAs (ceRNAs) which modulate the gene expression and maintain the functional balance of various gene networks (Kartha and Subramanian, 2014). Hence, we first identified the target genes of the lncRNAs in the signature and then put these genes into GO, KEGG, and REACTOM analysis. Results showed that target genes participate in biological processes, such as RNA stability and RNA splicing, and pathways such as RNA transport and spliceosome (Figure 5A,B). The PPI network of the target genes were shown in Figure 5C.

Moreover, genes, which was considered as related with the lncRNAs in the signature, were put into GO biological process and KEGG signaling pathways analysis. As shown in Supplementary Figure S2, lncRNA-related genes participate in biological processes such as cell proliferation and cell cycle. These data indicated the potential regulatory mechanism of lncRNAs in the signature.

## Discussion

Cervical cancer is a common gynecological malignancy with high mortality and its incidence of the younger trend in recent years. Due to the advancements in systemic chemotherapy and radiotherapy, the incidence of cervical cancer and mortality have dropped significantly. However, its recurrence rate and mortality in developing countries still remains high (Saslow et al., 2012). Considering the poor prognosis of cervical cancer, novel biomarkers, especially genetic biomarkers characterizing the inner molecular complexity, began to be used as predictors for the disease free survival and overall survival. With the development of high-throughput technology, more and more attention have been paid to the gene expression profiling which can be used to identify the biomarker that related to the heterogeneities and molecular of cervical cancer (Zhan et al., 2016; Dai et al., 2017). Previous studies have analyzed open-access datasets and establish an integrated model which could predict glioblastoma prognosis with high accuracy (Zhang et al., 2016). By applying the public breast cancer dataset, a series of recurrent copy number aberrations in complex patterns was also discovered via non-negative sparse singular value decomposition (Xi and Li, 2016). Here we downloaded and mined the open-access lnRNAs expression datasets of cervical cancer from GEO and performed a series analysis.

The microarray data we downloaded from GEO belongs to high-throughput biological data. In order to solve the common problem “curse-of-dimensionality” (small sample size combined with a very large number of genes) in high-throughput biological data, LASSO Cox regression model along with 10-fold cross validation was applied to analyze the expression data in the training cohort. As we have previously depicted, LASSO regression is good at handling high dimensional regression variables with no prior feature selection step by shrinking all regression coefficients toward zero, and thus forcing many regression variables to be exactly zero (Tibshirani, 1997; Mao et al., 2019a,b). It has been demonstrated that LASSO Cox regression model can achieves high stability and accurate predictions in dealing with the “curse-of-dimensionality” data (Algamal and Lee, 2015). By performing LASSO Cox regression model along with 10-fold cross validation, we identified a series of lncRNAs with the most powerful prognostic ability. At the same time, the regression coefficients were presented by LASSO Cox regression, based on which a nine-lncRNA signature for the prediction of disease free survival of cervical cancer was constructed.

In evaluating the predict accuracy of the nine-lncRNA signature, we first plotted the Kaplan–Meier curves and calculated the C-index of the signature. According to the results, significant difference was observed between the survive time along with status of the two groups separated by the same criteria in all three sets. Besides, further study showed that the nine-lncRNA signature has predict efficiency within different FIGO stage. Similarly, the C-index of lncRNA signature was higher than FIGO and a new variable combing both has the highest value than either alone. Moreover, we calculated the AUC for time specific ROC curves at continuous time point and dynamic AUC line was plotted to depict the temporal changes in accuracy. Results showed that the nine-lncRNA signature has a higher accuracy than FIGO stage alone. The new variable combined both nine-lncRNA signature and FIGO stage has better predict capacity for recurrence than either alone.

To gain more insights into the modulatory roles of the lncRNAs in the signature, we performed functional enrichment and annotation analysis for nine-lncRNAs in the signature. Results showed that lncRNAs in the signature might regulate biological processes, such as RNA splicing, cell proliferation and cell cycle, and pathways such as RNA transport and cell adhesion molecules. These data indicated the potential regulatory mechanism of lncRNAs in the signature.

Moreover, we also explored the regulatory mechanism of nine-lncRNAs in the signature by searching the published article. Among the nine-lncRNAs, lncRNA ATXN8OS participate in spinocerebellar ataxia by affecting the localization and activity of splicing factors and mutations in the ATXN8OS are associated with the amyotrophic lateral sclerosis (Moseley et al., 2006; Hirano et al., 2018). Dysregulation of lncRNA DIO3OS was closely related with inflammatory bowel disease (Wang et al., 2018). The downregulation of lncRNA EMX2OS might independently predict shorter recurrence-free survival of classical papillary thyroid cancer (Gu et al., 2018). LncRNA EMX2OS was also identified as associated with myalgic encephalomyelitis/chronic fatigue syndrome (Yang et al., 2018). LncRNA INE1 was considered as the potential hotspot for neurogenetic disorders (Thiselton et al., 2002). In Wilms’ tumors, reduced expression of lncRNA KCNQ1DN existing far from the H19/IGF2 region and may play regulatory role in tumor progression (Xin et al., 2000). LncRNA KCNQ1OT1 facilitates the progression of non-small-cell lung carcinoma via modulating miRNA-27b-3p/HSP90AA1 axis (Dong et al., 2018). On the other side, lncRNA KCNQ1OT1 controls maternal p57 expression in muscle cells by promoting H3K27me3 accumulation to an intragenic MyoD-binding region (Andresini et al., 2019). Studies on RFPL1S suggests that RFPL1S may function as a post-transcriptional regulation of the sense RFPL genes (Seroussi et al., 1999).

## Conclusion

In summary, we conducted comprehensive comparative analysis of lncRNA expression pattern and constructed a nine-lncRNA signature that can be applied to predict disease free survival in cervical cancer. Gene annotation and functional enrichment analysis further revealed the underlying mechanisms whereby lncRNAs in the signature exerts their biological roles in tumor progression. Although further study are still needed to confirm the established signature, our study here still provide valuable indication for both the basic research and clinical treatment of cervical cancer.

## Data Availability

The datasets generated for this study can be found in GEO (GSE44001) and TCGA.

## Author Contributions

YM contributed to the study design, data profiling, and manuscript draft. YZ and LD prepared the figures and tables. JD and XL performed the language editing. All authors reviewed and approved the final manuscript.

## Conflict of Interest Statement

The authors declare that the research was conducted in the absence of any commercial or financial relationships that could be construed as a potential conflict of interest.

## Footnotes

1 https://reactome.org/

## Supplementary Material

FIGURE S1 Box plots showed the distributions of genes for the 300 samples were nearly the same after normalization.

FIGURE S2 Functional enrichment analysis depicted the biological pathways and processes associated with correlated genes. The results of GO biological process enrichment **(A)** and KEGG signaling pathways analysis **(B)**.
