# Supplementary figures and images for: Prediction of Recurrence in Cervical Cancer Using a Nine-lncRNA Signature
Source: Front Genet. 2019 Apr 3;10:284. doi: 10.3389/fgene.2019.00284 (PMC6456668; doi:10.3389/fgene.2019.00284)

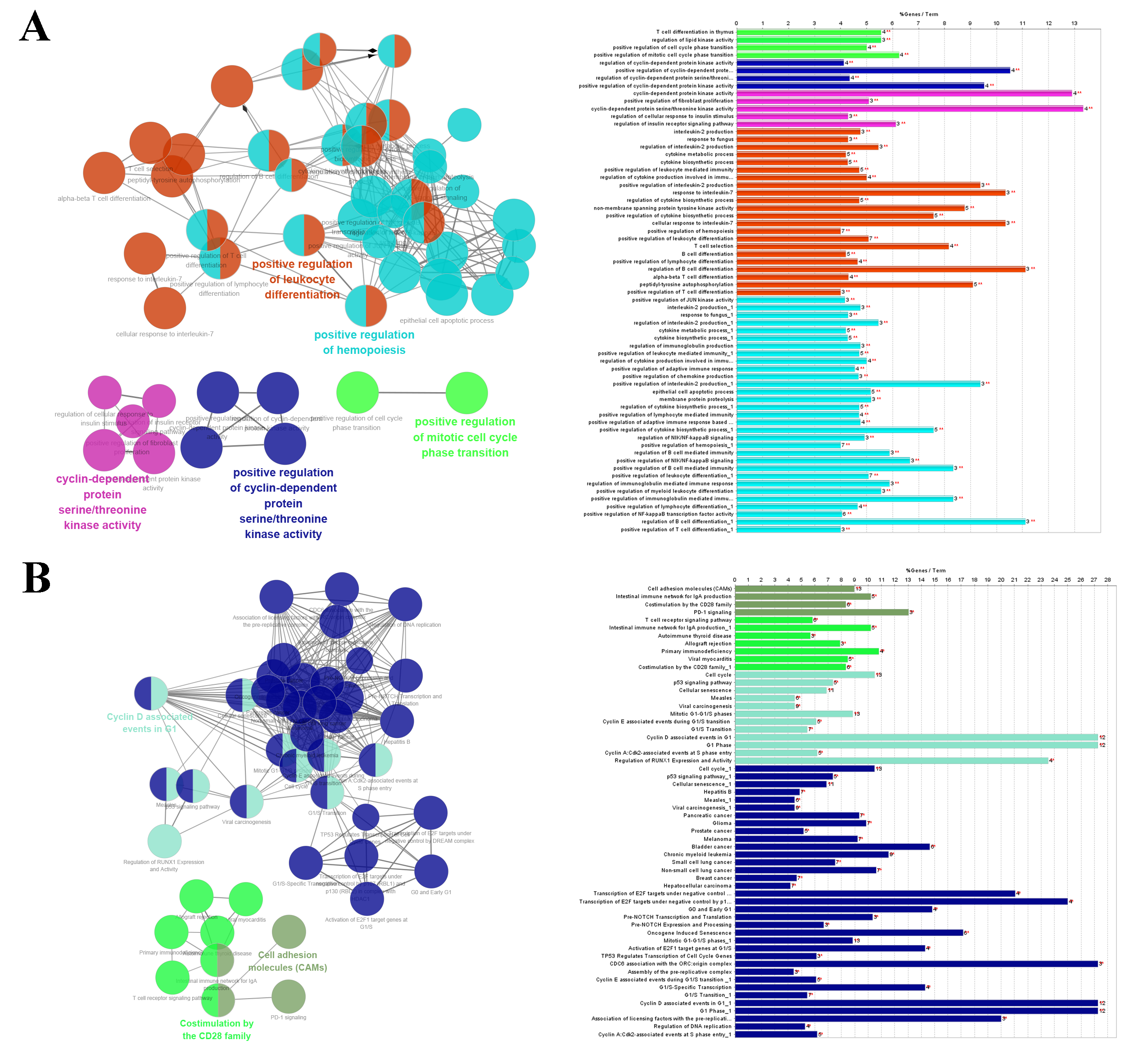

Supplement: FIGURE S2 — Functional enrichment analysis depicted the biological pathways and processes associated with correlated genes. The results of GO biological process enrichment (A) and KEGG signaling pathways analysis (B). [file Image_2.TIF]
